# Complications following an accidental sodium hypochlorite extrusion: A report of two cases

**DOI:** 10.4317/jced.50767

**Published:** 2012-07-01

**Authors:** María L. Bosch-Aranda, Carlos Canalda-Sahli, Rui Figueiredo, Cosme Gay-Escoda

**Affiliations:** 1 DDS. Fellow of the Postgraduate Oral Surgery and Orofacial Implantology, School of Dentistry, University of Barcelona (Spain).; 2 DDS, MD, PhD. Professor of Endodontics, School of Dentistry, University of Barcelona (Spain). Researcher of the IDIBELL Institute. Barcelona (Spain).; 3 DDS. Associate Professor of Oral Surgery. Professor of the Master of Oral Surgery and Orofacial Implantology. School of Dentistry of the University of Barcelona. Researcher of the IDIBELL Institute. Barcelona (Spain).; 4 DDS, MD, PhD. Chairman and Professor of Oral and Maxillofacial Surgery. Director of the Master of Oral Surgery and Orofacial Implantology. School of Dentistry of the University of Barcelona. Coordinator/Researcher of the IDIBELL Institute. Oral and Maxillofacial Surgeon of the Teknon Medical Center, Barcelona (Spain).

## Abstract

Sodium hypochlorite (NaOCl) is the most commonly used solution in root canal treatments, as it is a low-cost method that displays a very effective antimicrobial activity against microbiota of infected root canals. However, this solution can cause complications especially due to its cytotoxic features. When this solution is injected into the adjacent tissues, the patient usually experiences intense pain, and an urgent treatment should be implemented in order to prevent a long-term sequelae. This paper describes the clinical features of two patients that experienced an accidental extrusion of NaOCl after endodontic treatment of varying severity and with different treatments. Furthermore, it shows the long-term neurologic injuries that this type of accidents may cause and a treatment protocol for these situations will be suggested.

** Key words:**Nerve damage, root canal irrigation, root canal treatment, sodium hypochlorite.

## Introduction

Root canal treatment aims to the complete removal of the connective tissue and the destruction of residual microorganisms found in infected root canals. In addition, it seeks an effective seal in order to prevent recolonization of the root canal system with bacteria (1).

Irrigating solutions play a main role in the successful biomechanical preparation of root canals (2). In particular, sodium hypochlorite (NaOCl) is the most commonly used solution, as it is a low-cost method that displays a very effective antimicrobial activity against microbiota of infected root canals. Furthermore, the ability to oxidize and hydrolize cell proteins and its tissue solvent capacity, increase its value as an irrigant solution (3). Thus, this chemical agent reaches and cleans new areas within infected root canals, dissolving necrotic-purulent tissues. However, the cytotoxic effects are directly proportional to the concentration of the NaOCl (3,4). Complications causing severe tissue reactions associated with the accidental extrusion of NaOCl into periapical tissues have been described in the literature. Some authors have mentioned clinical situations where sodium hypochlorite was inadvertently injected into the maxillary sinus (6,7), or was unintentionally injected into the oral mucosa (5) causing adverse tissue reactions with life-threatening allergic responses (8,9).

In this paper, two cases involving the accidental extrusion of NaOCl during root canal irrigation are described, one of which present a persistent paresthesia in the left nasogenian area.

## Case Report 1

**Figure 1:**
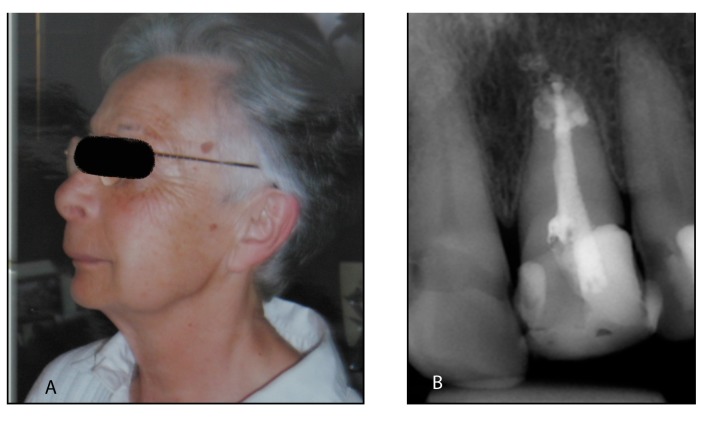
Case report nº1. (A) Lateral view of the patient 6 days after sodium hypochlorite extrusion of the left upper central incisor. (B) Periapical radiography of the affected incisor with periapical radiopaque areas, 1 year after sodium hypochlorite extrusion.

A 43 year-old female with no allergies and with a history of osteoarthritis, osteoporosis and Gilbert’s syndrome was seen in a dental clinic for implant-supported restoration of the maxillary anterior teeth. During the clinical examination, a lesion with a diameter of 0.5 cm, a whitish-yellow aspect, of soft elastic consistency located in the oral mucosa in the left upper central incisor area was observed. The patient reported an episode of sudden acute pain and severe swelling in the upper lip during a previous root canal treatment of the above-mentioned tooth that was carried out on January 2008. During this procedure, the NaOCl solution used for root canal irrigation was extruded into the adjacent tissues, probably due to a root resorption of the apical area, an apical foramen of great diameter or to an unintentionally injection into the foramen (solution concentration, gauge and length of irrigation needle remain unknown). Immediately, the dentist performed an abundant irrigation with sterile saline solution, administered a single intramuscular dose of sodium diclofenac (Voltaren® 75 mg., Novartis, Barcelona, Spain), and prescribed amoxicillin and clavulanic acid 875/125 mg (Augmentine® 875/125 mg, GlaxoSmithkline, Madrid, Spain), 1 tablet every 8 hours for 7 days. During the following days, the pain decreased although swelling persisted for 2 weeks (Fig. 1).

Four weeks following the accident, root canal instrumentation was completed, irrigation with sterile saline solution was performed and the canal was sealed by means of a lateral condensation technique using AH Plus™ root canal sealer (Dentsply DeTrey, Konstanz, Germany).

One year after the accident, the patient reported persistent pain in the apical area of the affected incisor that increased with pressure (Fig. 1). Periapical surgery was proposed to solve this complication but the patient denied any additional treatments and wanted to extract the tooth. Thus, dental extraction was performed followed by a histological exam of the associated lesion.

During the dental extraction, an apical root resorption, as well as, a buccal bone plate defect was observed. Following the elimination of the periapical lesion, the dentist removed the debris of the root canal sealing material. The anatomopathological study revealed fibrous tissue, fibrohematic material and unidentified birefringent material with no foreign body granulomatous reaction.

The patient complained of a persistent paresthesia located in the left nasogenian area with lateral rhinorrhoea which still persisted at the time of her last appointment, 2 years after the initial treatment and 9 months after the extraction.

## Case Report 2

**Figure 2:**
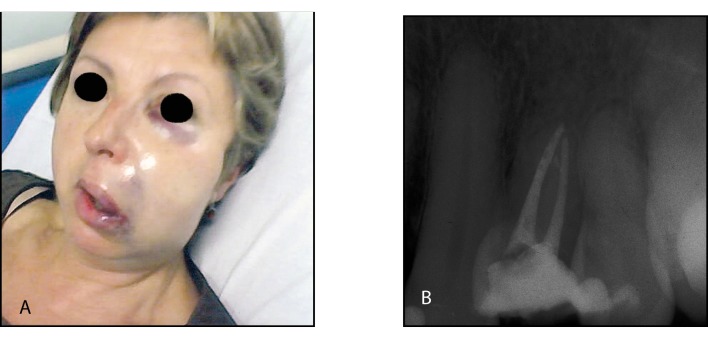
Case report nº2. (A) Frontal view of the patient following sodium hypochlorite extrusion in the upper left first premolar. (B) Periapical radiography of the affected premolar following root canal treatment.

A 53 year-old female was referred to the Emergency Department of the Hospital Universitario de Bellvitge (Barcelona, Spain) with a complaint of abrupt swelling of the left cheek following root canal therapy in the left upper first premolar. This treatment was carried out on August 2008 and was not completed due to severe pain reported during the procedure. The clinical examination showed an important swelling that comprised the area between the periorbital region and the mandibular angle, with hematoma formation in the infraorbital region. The oral mucosa didn’t show any type of necrosis (Fig. 2). Analgesics, corticoids and intravenous antibiotics were initially administered (dosage and administration route remain unknown).

Twenty-four hours after admission, the patient was discharged and showed some signs of recovery. Treatment consisted of a combination of amoxicillin and clavulanic acid 875/125 mg (Augmentine® 875/125 mg, GlaxoS-mithkline, Madrid, Spain) 1 tablet every 8 hours for 10 days; methylprednisolone (Urbason® 16 mg tablets, Sanofi Aventis, Barcelona, Spain) over a 6 day-period using a decreasing dosage; paracetamol (Xumadol® 1g soluble formulation, Kern Pharma, Barcelona, Spain) 1 tablet every 8 hours; omeprazole (20 mg tablets, Cinfa E.F.G, Navarra, Spain) 1 tablet every 24 hours, and local application of ice packs.

Ten days after the complication, the patient attended the Oral Surgery department at the Teknon Medical Center with persist edema and ecchymosis of the left cervical region, as well as, swelling in the second quadrant. A panoramic radiography was made to complete the examination.

After 2 weeks, the patient showed an important decreased of the ecchymosis and the radiographies failed to reveal any bone injuries.

Due to the improvement of the patient’s clinical situation, root canal treatment of the affected premolar was performed with working lengths of 18 mm for the vestibular canal and 17 mm for the palatal canal, both with an apical size of 50. In this case, irrigation was performed with a 2% chlorhexidine and EDTA solutions. Subsequently the canal was sealed following a lateral condensation technique using AH Plus™ root canal sealer. The final restoration was achieved using a metal-ceramic crown (Fig. 2).

Six months later a new panoramic radiography was taken and no significant alterations were identified. Furthermore, the clinical examination was normal and the patient did not show any symptoms.

Follow-up appointments (last one took place on July 2009) revealed no evidence of pathology in the upper left first premolar.

## Discussion

NaOCl is the most widespread irrigant used on root canal debridement. Used solutions may vary from 0.5% to 5.25% and its biocompatibility is inversely proportional to its concentration (10). When it comes in contact with vital tissues, NaOCl may cause haemolysis, skin ulceration, marked cell injury in endothelial cells and fibroblasts, and inhibition of neutrophil migration (11). Thus, several studies have been carried out in order to compare the cytotoxic effects with those of other irrigant solutions. Regarding this aspect, Navarro-Escobar et al. (12) compared the cytotoxicity of 15% citric acid, 5% phosphoric acid and 2.5% NaOCl. They concluded that the irrigating solution with the highest percentage of cell viability was 2.5% NaOCl at both 0.1% and 0.5% dilu-tions. Furthermore, Vianna et al. (13) performed an in-vitro investigation on the antimicrobial activity of 0.2%, 1%, and 2% chlorhexidine gluconate (gel and liquid) against endodontic pathogens and compared the results with the ones achieved by 0.5%, 1%, 2.5%, 4%, and 5.25% sodium hypochlorite. The authors concluded that all tested irrigants eliminated all microorganisms. However, this report remarks an important disadvantage of chlorhexidine gluconate as it does not dissolve organic tissues found in root canals. Therefore chlorhexidine gluconate is recommended as an alternative irrigating solution to NaOCl, especially in cases of open apex, suspected allergies to NaOCl or in the event of accidental extrusion (13).

Most of the complications associated with an accidental sodium hypochlorite extrusion refer to irrigation needle injection into the canal. Several measures can be implemented to avoid this situation. To begin with, irrigation with NaOCl should be avoided in the apical region if the working lengths have not been previously measured. Once all working lengths have been determined, it is recommended to place a positioning rubber stops on the irrigation needles, preferable with lateral exit, to prevent pressure during irrigation and accidental injection. Furthermore, the operator should ensure that irrigation is performed under low and constant pressure to prevent leakage of the solution to the root canal.

When NaOCl is accidentally injected into periapical tissues, most authors describe similar clinical signs and symptoms (5-9,11,15,16). First of all, the initial painful swelling may spread to adjacent tissues. In addition it may reach the periorbital area, the upper lip, the cheek and can be accompanied by profuse interstitial bleeding with haemorrhage of the skin and mucosa. If the irrigant has leaked into the maxillary sinus, the patient may report a chlorine taste and irritation of the throat. Finally, necrosis and secondary infection may become evident and other severe complications like anaesthesia or paresthesia may be diagnosed.

Most studies describe several therapies depending on the nature and seriousness of the accident but there is no well-established treatment protocol for such cases. Therefore, we propose several guidelines in the event of ac-cidental extrusion of NaOCl ( Table 1). In moderate cases, an outpatient therapy is usually recommended. However, in life-threatening situations or when a serious infection of adjacent tissues is expected, admission to a hospital should be considered in order to prescribe intravenous medication, as described in case nº 2. Nevertheless, there is some controversy on this subject, as some authors (16) always recommend aggressive measures such as the use of antibiotics and intravenous anti-inflammatory medication. Hospital admission may be advisable to allow a close monitoring and improve clinical outcomes of these patients.

**Table 1:**
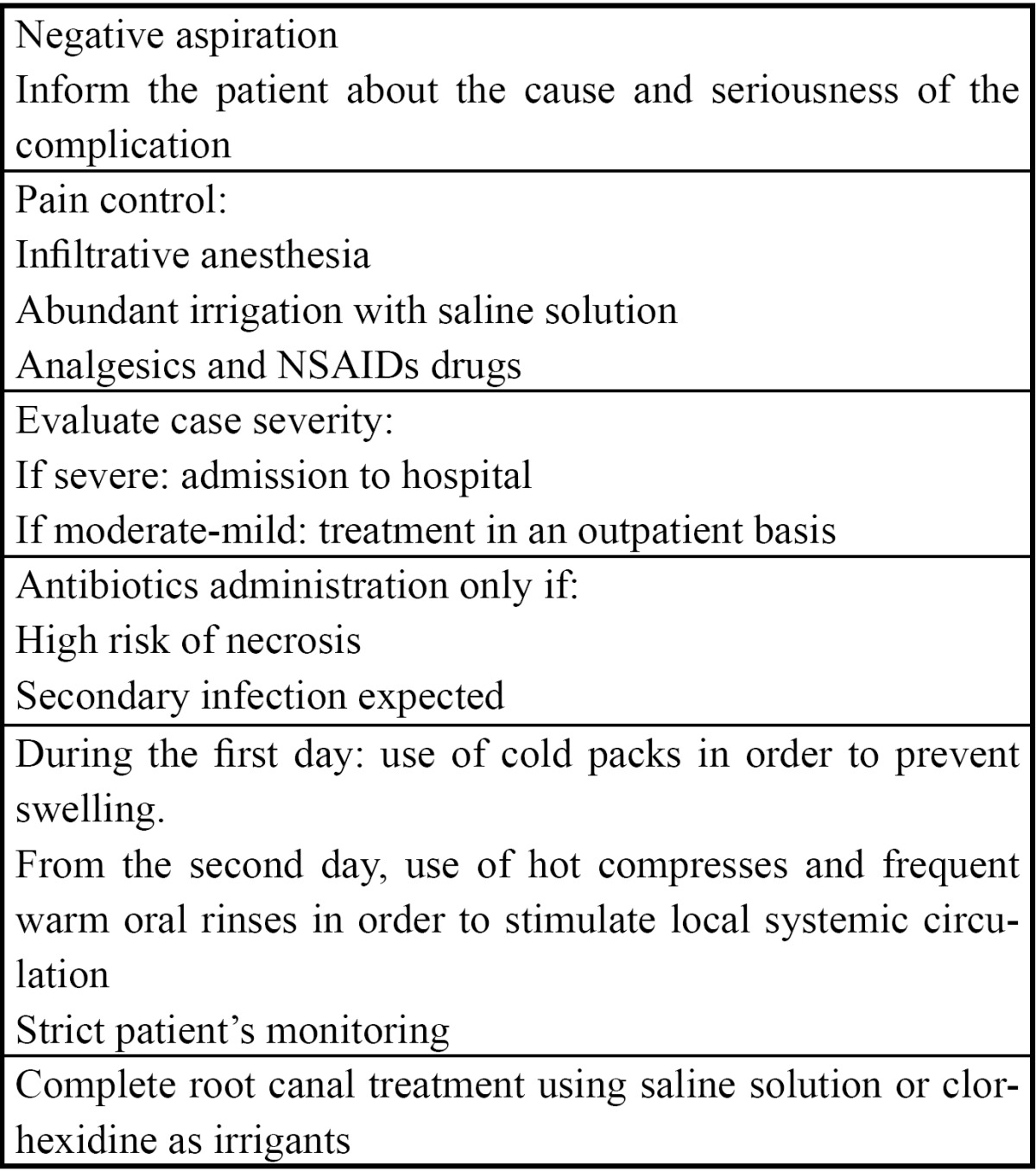
Treatment guidelines after accidental extrusion of NaOCl.

The main short-term objective is to treat the intense and sudden onset pain suffered by the patient. Thus, it may be necessary to perform reanaesthesia of the affected area and prescribe analgesics. Additionally, as a consequence of the great oedema of the adjacent tissues, both steroidal and non-steroidal anti-inflammatory drugs are commonly used, although no studies have been published supporting this treatment. Furthermore, additional measures can be recommended, such as the use of cold packs during the first day in order to prevent swelling or the use of hot compresses, starting after the second day, in order to stimulate local systemic circulation (15).

The routine use of antibiotics is controversial. Antibiotics should be administered only if there is any clinical evidence of wound infection or if necrosis is expected (5).

Finally, root canal treatment can be completed, although- as previously stated- irrigation with a sterile saline solution or with chlorhexidine gluconate are highly recommended (0.2%-2%) (13,14,17).

This is not the first time that neurological injuries are associated with this complication, although most of the times it is reversible. In our first case report, the patient presented a persistent paresthesia probably due to the chemical injury of the affected area.

Nerve chemical injuries are related to the toxicity of the substance and the easiness to remove it (18). Therefore, the most suitable concentration of NaOCl for endodontic irrigation may be 0.5 or 1% with the pH close to neutral, obtaining an optimal antimicrobial effect with minimal tissue irritating injury (19). However different concentrations of NaOCl (e.g., 2.5 %) are currently used as root-canal irrigants in order to eliminate facultative an-aerobic and strict anaerobic bacteria.

In the event of accidental extrusion of NaOCl into the periapical area, the solution should be removed as soon as possible, performing a negative aspiration with the same irrigation syringe. Then irrigation of the area with abundant sterile saline solution should be made. By doing this, the exposure time of the nerve to the irrigant agent is reduced. In addition, it is important to identify the affected nerve branches since inferior alveolar nerve damage may lead to an important area of sensitivity impairment, whereas, when small nerve fibres are injured the effect is more limited, as in our first case report.

Finally, microsurgical repair may be considered, as it provides an improvement of the neurosensory function of patients that present nerve injuries. However, the results published up to date show unpredictable results (20), indicating that this kind of surgery should only be indicated when the patient’s quality of life is significantly affected.

## Conclusions

Based on the presented case reports, special attention must be drawn to the potential risks associated with the use of NaOCl as an irrigant for root canal therapy. Thus, it is important to carry out an effective technique in order to avoid complications. In the event of accidental extrusion of NaOCl, treatment guidelines should be applied according to the magnitude of each individual case.
